# Biophotopol: A Sustainable Photopolymer for Holographic Data Storage Applications

**DOI:** 10.3390/ma5050772

**Published:** 2012-05-02

**Authors:** Manuel Ortuño, Sergi Gallego, Andrés Márquez, Cristian Neipp, Inmaculada Pascual, Augusto Beléndez

**Affiliations:** 1 Departamento de Física, Ingeniería de Sistemas y Teoría de la Señal, Universidad de Alicante, Apartado 99, Alicante E03080, Spain; E-Mails: sergi.gallego@ua.es (S.G.); andres.marquez@ua.es (A.M.); cristian@dfists.ua.es (C.N.); a.belendez@ua.es (A.B.); 2 Instituto Universitario de Física Aplicada a las Ciencias y las Tecnologías, Universidad de Alicante, Apartado 99, Alicante E03080, Spain; 3 Departamento de Óptica, Farmacología y Anatomía, Universidad de Alicante, Apartado 99, Alicante E03080, Spain; E-Mail: pascual@ua.es

**Keywords:** holographic materials, photopolymers, low toxicity

## Abstract

Photopolymers have proved to be useful for different holographic applications such as holographic data storage or holographic optical elements. However, most photopolymers have certain undesirable features, such as the toxicity of some of their components or their low environmental compatibility. For this reason, the Holography and Optical Processing Group at the University of Alicante developed a new dry photopolymer with low toxicity and high thickness called biophotopol, which is very adequate for holographic data storage applications. In this paper we describe our recent studies on biophotopol and the main characteristics of this material.

## 1. Introduction

Photopolymers are light sensitive materials with adequate characteristics for application as recording material in new devices such as optical elements, holographic memories or as holographic recording media in research. A specific set of parameters is required of the photopolymers: good energetic sensitivity in order to save energy during the recording process, an adequate spectral sensitivity, and a high resolution to enable fidelity reproduction of the stored information. Additionally, new trends in photopolymers include better environmental compatibility, low toxicity, ease of production and good recycling properties. We describe our line of research on this subject and the main results obtained with biophotopol, a new type of photopolymeric material that the Holography and Optical Processing research team at the University of Alicante is developing [1,2,3].

Usually, photopolymers have a photoinitiator system that absorbs light and generates free radicals that initiate the radical polymerization reaction of one or various monomers. In the case of holographic recording, the basic mechanism of hologram formation involves modulation of the refractive index between polymerized and non-polymerized zones, corresponding to the “bright” and “dark” zones respectively, in the diffraction grating generated due to the interference of the recording beams [4].

There are many types of photopolymers, which can be differentiated by the type of binder, since this component determines to a great extent the choice of monomer, dye and initiator used in the photopolymer. Examples of photopolymers with a hydrophobic binder are polyesters of methacrylic acid, which contain acrylic ester type monomers. Photopolymers with a hydrophilic binder include hybrid materials made using the sol-gel procedure. Photopolymers with a poly (vinyl alcohol) (PVA) or gelatine binder and acrylamide (AA) based monomers are also hydrophilic. All these photopolymers have an undesirable feature, the high toxicity of their components and their low environmental compatibility, particularly if we analyse the life cycle of the devices made with these materials and their interaction with the environment. The environmental compatibility and life cycle are important features that must always be considered when developing new materials [5].

Hydrophilic photopolymers with AA as the polymerizable monomer are versatile materials for use as holographic recording media. Our research team optimized these photopolymers in layers up to 1 mm thick and they were used to obtain holograms with high diffraction efficiency and good energetic sensitivity. These photopolymers have good properties, in particular, their good energetic sensitivity compared with that of other available materials; the possibility of easily adapting their spectral sensitivity to the type of recording laser used by simply changing the sensitizer dye; high diffraction efficiency, together with an acceptable resolution and signal/noise ratio [6].

The high thickness of the 1 mm PVA/AA photopolymer facilitates its possible use in WORM (write once read many) type holographic memories. Using a specific holographic set-up, we multiplexed data pages on this material and determined the response of the photopolymer in terms of different holographic parameters: diffraction efficiency, sensitivity, bit error rate and angular response [7].

The main drawback of an AA-based photopolymer as far as the environment is concerned is the acrylamide, a substance which has been known to be carcinogenic for many years. Recent investigations confirm its toxic potential [8,9].

In order to develop a new photopolymer with environmental compatibility we used the 1 mm PVA/AA photopolymer as a reference and evaluated its most environmentally unfriendly components in order to propose alternatives. We developed a new photopolymer called biophotopol with photochemical and holographic features similar to those of AA-based materials but with an improved design from the environmental point of view. We obtained quantitative values of: shrinkage, polymerization rate, polymer refractive index and relation between polymerization and recording intensity [10].

## 2. Experimental Section

The standard photopolymer (PA, Table 1) is composed of AA as polymerizable monomer, triethanolamine (TEA) as coinitiator and plasticizer, yellowish eosin (YE) as dye, PVA as binder and a small proportion of water as additional plasticizer. It may also contain *N*,*N*’-methylene-bis-acrylamide (BMA) as crosslinking monomer.

AA and BMA are toxic monomers, the former more so than the latter. YE also poses problems due to the four Br atoms in its molecule. The decomposition by-products of YE are not photoactive but they can interact with the environment to generate halogenated substances, which are toxic. All dyes derived from fluorescein have these characteristics, such as Bengal rose or erytrosin B dyes, commonly used in hydrophilic AA-based photopolymers. As evidence of their toxicity, we can mention the research being done on their possible application as pesticides [11,12].

In biophotopol we use the sodium salt 5’-riboflavin monophosphate (PRF) as dye, bearing in mind that this substance is water-soluble and exists in the environment, so it is not likely to cause environmental problems.

With the photopolymer PA the highest absorption takes place in the 485–550 nm region, whereas in the case of the photopolymer with PRF, the main absorption takes place at wavelengths lower than 500 nm. The argon laser used in the hologram recording experiments was tuned at 514 nm where the absorption for photopolymer PA is higher than that for photopolymers with PRF, T = 0.4% and T = 46.7% respectively.

**Table 1:** Composition of the prepolymer solution in molarity, poly (vinyl alcohol) (PVA) in percentage.

	PA	PB	PC	PD	PE	PF
AA	0.34	–	–	–	–	–
NaAO	–	0.34	0.34	0.34	0.34	0.31
YE	9.0 × 10^−5^	–	–	–	–	9.0 × 10^−5^
PRF	–	1.0 × 10^−3^	1.0 × 10^−3^	1.0 × 10^−3^	1.0 × 10^−3^	–
TEA	0.15	0.15	0.15	9.2 × 10^−3^	9.2 × 10^−3^	0.15
PVA	13.4%	13.4 %	13.4 %	13.4 %	13.4 %	13.4 %
DHEBA	–	–	6.4 × 10^−3^	–	6.4 × 10^−3^	13.0 × 10^−3^

For monomer substitution it is necessary to use another vinyl monomer that is less toxic than AA. We used sodium acrylate prepared in situ by means of a one-pot reaction with acrylic acid and sodium hydroxide in the prepolymer solution used to prepare the layers. The toxicity of sodium acrylate is lower than that of AA. We replaced AA by NaAO and YE by PRF in the photopolymer PB.

### 2.1. Crosslinking

BMA, a known reproductive toxicant, is the crosslinker usually used in a standard AA-based photopolymer. We used *N*,*N*’-(1,2-dihydroxyethylene) bisacrylamide (DHEBA) in biophotopol as an alternative to BMA. DHEBA has occasionally been used in hydrophilic photopolymers due to its good solubility in water. This molecule is suitable for the new photopolymer because its two hydroxyl groups are compatible with the structure of sodium polyacrylate generated in the photopolymerization. In this manner, hydrogen bonds may be formed with the PVA binder and TEA and water plasticizers. Although there are no studies in the bibliography that suggest this substance is toxic, future research may show it has a certain level of toxicity; however, it would be less toxic than BMA. Photopolymer PC contains NaAO, PRF and DHEBA.

### 2.2. Improving the Performance of Biophotopol

PRF can generate radicals without a coinitiator but a low content of TEA is necessary in order to improve the performance of the material. Photopolymer PE contains DHEBA whereas photopolymer PD does not, and both of them have a low TEA content. Photopolymer PF contains DHEBA and the standard initiation system with YE and TEA for comparison.

### 2.3. Preparation of the Photopolymers

Biophotopol has a hydrophilic binder like the AA-based standard photopolymer and this implies that during its production the main solvent used is water. Biophotopol does not use any additional cosolvent. Any products and devices made with this photopolymer could also be eliminated, once their useful life was over, by dissolving in water. Therefore, this material has an advantage over hydrophobic photopolymers because it avoids the use of petroleum-based solvents, which are toxic and flammable.

The solutions, whose compositions can be seen in Table 1, with water as the solvent are deposited, using the force of gravity, in polystyrene moulds, and left in the dark (RH = 40–45%, T = 20–23 °C). When part of the water has evaporated (after about 6 days), the layer has enough mechanical resistance and can be extracted from the mould without deforming. The solid film is cut into 900 μm thick squares and adhered, without adhesive, to the surface of glass plates measuring 6.5 × 6.5 cm^2^. The plates are then ready for exposure, which takes place immediately.

### 2.4. Holographic Set-Up

**Figure 1:**
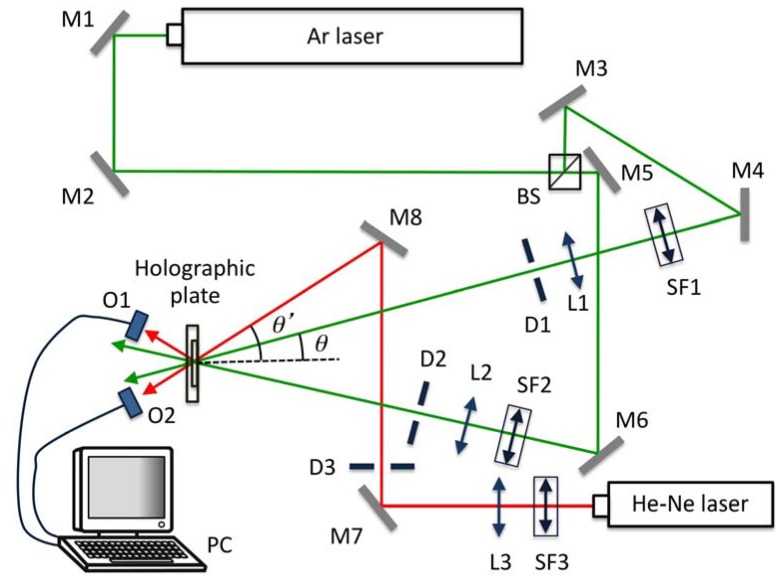
Experimental set-up. BS: Beamsplitter, Mi: mirror, SFi: spatial filter, Li: lens, Di: diaphragm, Oi: optical power meter, PC: data recorder.

To study the behaviour of these photopolymers as a holographic recording material, we obtained diffraction gratings using a holographic setup. The experimental device is shown in Figure 1. An Argon laser tuned at a wavelength of 514 nm was used to store diffraction gratings by means of continuous laser exposure. The laser beam was split into two secondary beams with an intensity ratio of 1:1. The diameter of these beams was increased to 1.5 cm with an expander, while spatial filtering was ensured. The object and reference beams were recombined at the sample at an angle of 16.8° to the normal with an appropriate set of mirrors, and the spatial frequency obtained was 1125 lines/mm. The working intensity at 514 nm was 5 mW/cm^2^. The diffracted and transmitted intensity were monitored in real time with a He-Ne laser positioned at Bragg’s angle (20.8°) tuned to 633 nm, where the material does not polymerize. In order to obtain transmission and diffraction efficiency as a function of the angle at reconstruction we placed the plates on a rotating stage. The diffraction efficiency (DE) was calculated as the ratio of the transmitted and diffracted beam, respectively, to the incident power.

### 2.5. Basic Characterization of Biophotopol

In holographic applications it is very difficult to directly measure the parameters necessary for photopolymers to be used as optical recording media due to two different phenomena: polymer formation and monomer diffusion. We propose two measuring techniques. One of them is a direct method based on zero spatial frequency recording to eliminate the effect of diffusion, and the other one is based on interferometric techniques, both in transmission and in reflection, to obtain quantitative values of: shrinkage, polymerization rate, polymer refractive index and relation between polymerization and recording intensity.

To analyze and characterize the different compositions at the zero spatial frequency limit we used the equations described in [13]. To generate monomer polymerization at the zero spatial frequency limit we used a wavelength of 532 nm with an intensity of 1 mW/cm^2^ (at this wavelength the dye shows maximum absorption), and for the interferometric arm, we used a He-Ne laser to generate the interference pattern, since the photopolymer does not show any absorption at 633 nm [14]. We implemented a Young’s fringe based two beam interferometer. This interferometer has been successfully applied in the phase-shift characterization of liquid crystal displays (LCDs). The thickness of these samples was around 100 μm. The compositions analyzed are shown in Table 2.

**Table 2:** Solution composition and thickness of the layers for basic characterization of biophotopol.

	Composition A	Composition B	Composition C	Composition D	Composition E
NaAO (g)	0.75	1.5	0.75	1.5	0.75
BMA (g)	–	0.1	0.2	–	–
H_2_O (mL)	12.5	12.5	12.5	12.5	12.5
TEA (mL)	1.25	1.5	1.5	3	3
PVA (mL) (15% w/v)	12.5	12.5	12.5	12.5	12.5
YE (mL) (0.8% w/v)	0.6	0.6	0.6	0.6	–
PRF (g)	–	–	–	–	0.14
Thickness ± 4 (μm)	105	100	97	107	106

#### 2.5.1. Transmission Experiments

**Figure 2:**
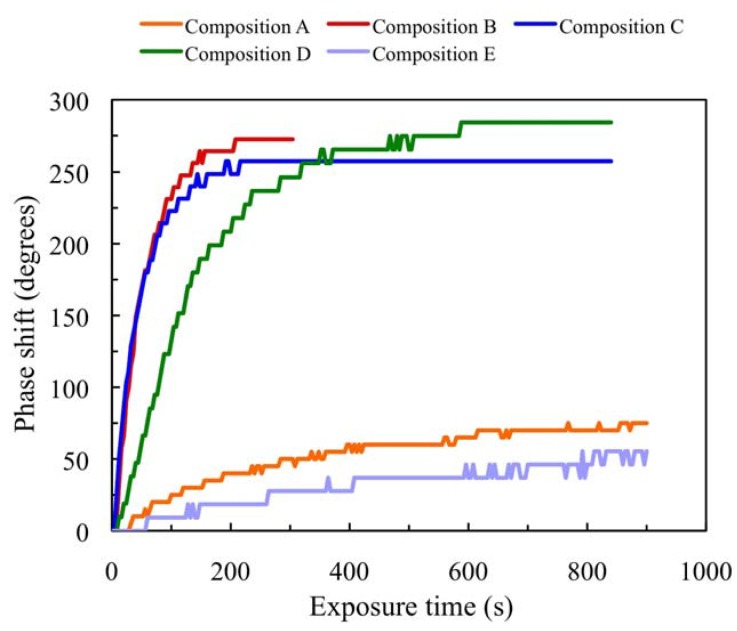
Phase shift as a function of exposure time in transmission experiments for photopolymer compositions A, B, C, D and E.

In the transmission analysis, the effects of refractive index changes and thickness variation during polymerization can be seen. The results for the five compositions in Table 2 are presented in Figure 2. As can be seen, the phase changes between exposed and non-exposed zones for compositions A and E are very weak (around 60°); therefore the polymerization rate is low. As the velocity of the reaction is very slow, after 1000 s compositions A, E and D (compositions without crosslinker) continue to change. Compositions B and C (compositions with crosslinker) show very similar behaviour in the transmission analysis. In these compositions, after 400 s the reaction stops and the layers are in the saturation state (where the phase shift does not change with illumination). This fact indicates that all the monomer is consumed in the illuminated zones. Moreover, it is important to note that the phase shift is less than 360° and this phase depth is required for many diffractive applications (*i.e.*, to manufacture lenses). To achieve higher phase depth the thickness of the layer should be increased. Furthermore, it is important to mention the influence of the TEA concentration when comparing layers B and D. These two compositions have the same quantity of NaAO; in addition, composition B has a crosslinker but the phase shift achieved is similar. This fact can be explained by the differences in TEA concentration. As composition B has a low content of TEA it is more difficult for the NaAO to dissolve and it can crystallize during the drying and recording process.

#### 2.5.2. Reflection Experiments

**Figure 3:**
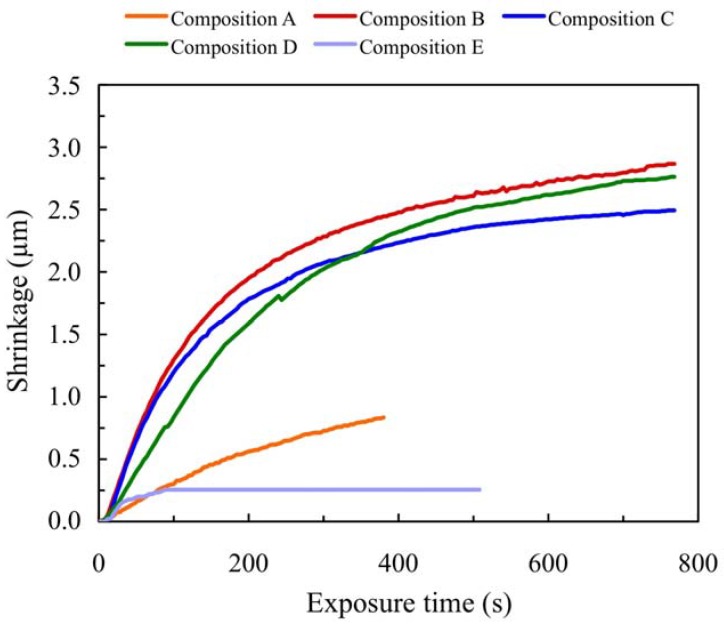
Shrinkage as a function of exposure time for photopolymer compositions A, B, C, D, and E in reflection experiments.

Using reflection experiments we can obtain the thickness variation for different compositions (with and without crosslinker, with different monomer and TEA concentrations), at zero frequency. Once the phase shift between exposed and non-exposed zones has been obtained as a function of exposure with the reflection interferometer, the shrinkage of the layer during exposure can be directly calculated. In Figure 3 we present the results obtained in the reflection analysis for NaAO based compositions (A, B, C, D and E). As can be seen, the behaviour is similar to that observed in the transmission analysis. It is important to note that compositions B, C and D behave similarly. These layers can achieve around 3000° of phase shift. For composition E, for which PRF is the dye, the phase shift is around 300° due to the poor solubility inside the photopolymer and the low monomer reaction velocity. Figure 3 shows shrinkage values between 0.5% and 3% for 100 μm photopolymer layers.

## 3. Results and Discussion 

**Figure 4:**
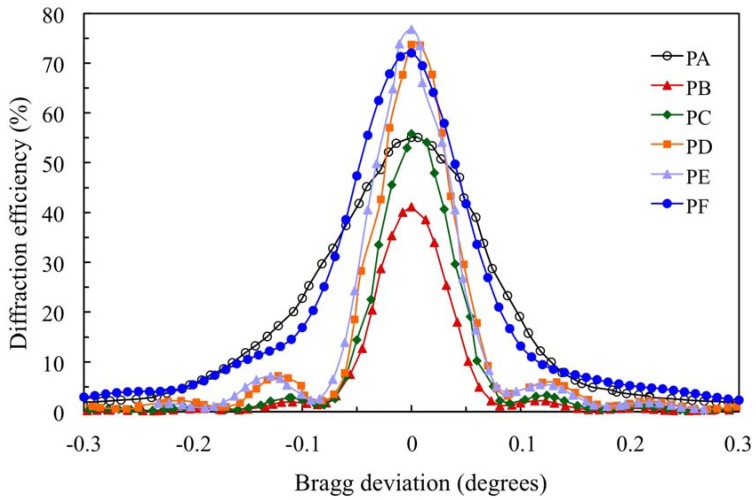
Angular scan for the holograms recorded in the 900 μm photopolymer layers.

Figure 4 shows the angular scan for the transmission gratings recorded in the 900 μm photopolymer layers for which the composition is shown in Table 1.

The standard photopolymer PA with AA and YE/TEA has a maximum diffraction efficiency (DE_max_) of 55%. We can obtain a less toxic photopolymer with YE substituting AA by NaAO and BMA by DHEBA. A good result with DE_max_ = 72% is obtained for photopolymer PF. Substitution of AA and YE in the photopolymer PB yields a low DE_max_ (41%). Adding the crosslinker DHEBA in photopolymer PC increase the DE_max_ to 56%, which is similar to the Demax of the standard photopolymer PA. Optimization of the TEA concentration in biophotopol improves the performance with DE_max_ = 74% for photopolymer PD. Additionally, using DHEBA in photopolymer PF makes it possible to obtain a higher diffraction efficiency DE_max_ (77%).

We fit the experimental data with the Kogelnik coupled wave theory and we obtained these values for the index modulation n_1_ and the attenuation coefficient α (μm^−1^). The detailed equations can be seen in [15,16]: PA (n_1_ = 23.0 × 10^−5^, α = 4.5 × 10^−3^), PB (n_1_ = 17.7 × 10^−5^, α = 1.2 × 10^−3^), PC (n_1_ = 22.5 × 10^−5^, α = 1.0 × 10^−3^), PD (n_1_ = 29 × 10^−5^, α = 1.0 × 10^−3^), PE (n_1_ = 31 × 10^−5^, α = 1.0 × 10^−3^), PF (n_1_ = 29.0 × 10^−5^, α = 3 × 10^−3^). The photopolymers PA and PF with the dye YE have an attenuation coefficient higher than photopolymers with PRF.

### 3.1. Parameter Estimation

Taking into account the experimental results of the previous two sections our aim was to obtain some basic parameters of the biophotopol compositions for thin layers (around 100 μm thick). Shrinkage is due to layer compaction during polymerization. To determine the velocity of the chemical reaction, the polymerization rate, FR, we used Equation (13) presented in [13]. The FR results for each composition are shown in Table 3. The correlation value of the fittings indicates that there is good agreement between theory and experiments. In addition, compositions A and D have similar values of FR because the only difference between them is the monomer concentration. We have omitted the fitting for composition F due to the poor repeatability. The problem with the 100 μm layers is that the solubility of PRF inside the layer depends dramatically on the drying conditions.

The values for *n_p_* are important when compared with the monomer values; *i.e.*, the refractive index of acrylamide is clearly lower than that of NaAO. To determine these values we followed the steps indicated in [17]. Furthermore, the most important point is to compare *n_m_* with *n_p_* and these parameters with *n_b_*. In this sense the importance of a crosslinker to increase *n_p_* was demonstrated using the zero frequency technique*.* The low value obtained for composition C can be explained by the precipitation of part of the BMA during the drying process. To improve this composition the concentration of TEA should be increased as in composition D and the compositions described in Ref. [17]. As can be seen in Table 4, the value of *n_p_* for composition A is lower than for composition D. This can be explained by the higher concentration of monomer, which leads to longer polymer chains (larger values of refractive index).

**Table 3:** Polymerization rates (*F_R_*) and correlation parameter *R*^2^ for the fittings obtained for each chemical composition.

	Composition A	Composition B	Composition C	Composition D
*F_R_*x10^−3^ (s^−1^)	3.2	3.6	4.1	3.3
*R*^2^	0.999	0.993	0.992	0.994

**Table 4:** Initial monomer volume fraction, polymer refractive index, *n*_p_, and binder refractive index, *n_b_*, for each composition based on Biophotopol.

	Composition A	Composition B	Composition C	Composition D	Composition E
Initial monomer volume fraction	0.13	0.15	0.25	0.17	0.11
*n_p_*	1.578	1.603	1.585	1.595	1.51
*n_b_*	1.470	1.476	1.476	1.474	1.470

**Figure 5:**
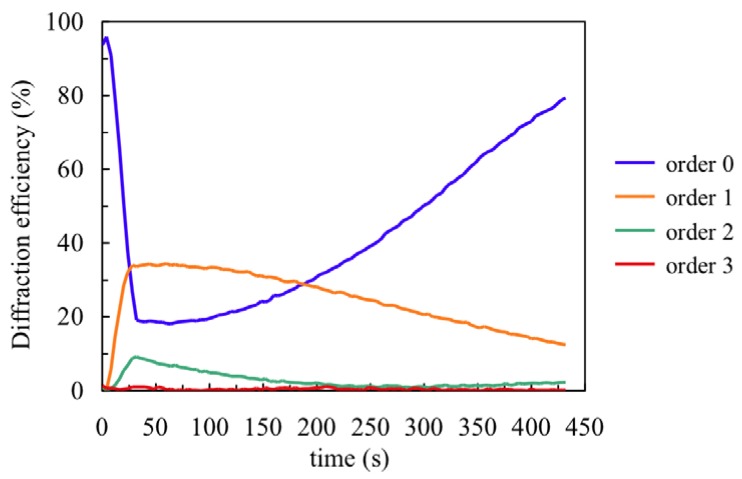
Fourth first reflected orders for composition A.

Once the chemical parameters have been determined, the monomer diffusion can be fitted recording very low spatial frequency gratings. To determine the NaAO diffusion inside the material we measured the first eight diffracted orders (reflected in this case) during recording (30 s) and the dark evolution of the diffraction efficiencies after recording. Figure 5 shows the behaviour for a recording of spatial period 0.168 μm and the post-exposure evolution. Following the steps described in [18] we obtained the surface profile of the layer by fitting the experimental DE data with a Fermi-Dirac function. It is well known that during recording the illuminated photopolymer zones shrink due to polymerization and subsequently swell due to monomer diffusion from the dark zones. The velocity of swelling depends on the material diffusivity, *D*, the diffusion time, *τ*, and the grating period, Λ:

(1) $$D=\frac{\Lambda^{2}}{4\pi^{2}\tau }$$

Using the data from Figure 5 we obtain the following values: *τ* = 5000 s and *D* = 1.4 × 10^−9^ cm^2^ s^−1^. Once the polymerization rates and monomer diffusion inside the material are known, we can apply the holographic diffusion models to simulate hologram formation in these photopolymers [19].

## 4. Conclusions 

We developed a new photopolymer, biophotopol, with higher environmental compatibility than that of standard acrylamide based photopolymers. We studied the main holographic parameters of biophotopol and obtained good performance in 900 μm layers, similar to that of the standard layers. High diffraction efficiencies (DE_max_ = 77%) with an energetic exposure of 200 mJ/cm^2^ were obtained. We observed some properties of biophotopol that could be useful for recording many holograms by multiplexing: the small angular interval of the angular response curve (0.2°). In addition, the by-products derived from PRF after photodecomposition are also dyed and can initiate new polymerization reactions with a similar efficiency to that of the initial PRF molecule. Therefore, the dye is not a limiting factor, especially when many holograms are recorded. This is the opposite of what happens in the case of the standard photopolymer in which the by-products of the reaction of YE and TEA are colourless. Thus, in a multiplexing recording the dye concentration decreases as each new hologram is recorded and the YE content is a limiting factor, with the result that the thickness of the polymer grating in each hologram varies. Additionally, we obtained the main parameters related to the kinetics of the photopolymerization reaction. Therefore, this photopolymer is an alternative to traditional acrylamide based photopolymers for use as holographic recording material in holographic optical elements and data storage applications.

## References

[1] Samui A.B. (2008). Holographic recording medium. Rec. Pat. Mater. Sci..

[2] Lessard R.A. (1997). Polymer used as holographic recording materials: A review. Proc. SPIE.

[3] Ortuño M., Gallego S., García C., Neipp C., Pascual I. (2003). Holographic characteristics of a 1 mm thick photopolymer to be used in holographic memories. Appl. Opt..

[4] Kveton M., Fiala P., Havránek A., Rosen J. (2011). Polymer holography in acrylamide-based recording material. Holography, Research and Technologies.

[5] Anastas P.T., Warner J.C. (2000). Green Chemistry: Theory and Practice.

[6] Ortuño M., Gallego S., García C., Neipp C., Beléndez A., Pascual I. (2003). Optimization of a 1 mm thick PVA/acrylamide recording material to obtain holographic memories: Method of preparation and holographic properties. Appl. Phys. B.

[7] Fernández E., García C., Pascual I., Ortuño M., Gallego S., Beléndez A. (2006). Optimization of a thick polyvinyl alcohol-acrylamide photopolymer for data storage using a combination of angular and peristrophic holographic multiplexing. Appl. Opt..

[8] Hashimoto K., Aldridge W.N. (1970). Biochemical studies on acrylamide, a neurotoxic agent. Biochem. Pharmacol..

[9] Mendel F. (2003). Chemistry, biochemistry, and safety of acrylamide. A review. J. Agric. Food. Chem..

[10] Ortuño M., Fernández E., Gallego S., Beléndez A., Pascual I. (2007). New photopolymer holographic recording material with sustainable design. Opt. Express.

[11] Lipman A.L. (1995). Safety of xanthene dyes according to the U.S. Food and Drug Administration. Light-Activated Pest Control. ACS Symp. Ser..

[12] Heitz J.R. (1982). Insectic Mode Action.

[13] Gallego S., Márquez A., Ortuño M., Marini S., Francés J. (2011). High environmental compatibility photopolymers compared to PVA/AA based materials at zero spatial frequency limit. Opt. Mat..

[14] Gallego S., Márquez A., Méndez D., Ortuño M., Neipp C., Fernández E., Pascual I., Beléndez A. (2008). Analysis of PVA/AA based photopolymers at the zero spatial frequency limit using interferometric methods. Appl. Opt..

[15] Gallego S., Ortuño M., Neipp C., Márquez A., Beléndez A., Pascual I., Kelly J.V., Sheridan J.T. (2005). 3 Dimensional analysis of holographic photopolymers based memories. Opt. Express.

[16] Neipp C., Sheridan J.T., Gallego S., Ortuño M., Márquez A., Pascual I., Beléndez A. (2004). Effect of a depth attenuated refractive index profile in the angular responses of the efficiency of higher orders in volume gratings recorded in a PVA/Acrylamide photopolymer. Opt. Comm..

[17] Kelly J.V., Gleeson M.R., Close C.E., O’Neill F.T., Sheridan J.T., Gallego S., Neipp C. (2005). Temporal analysis of grating formation in photopolymer using the nonlocal polymerization-driven diffusion model. Opt. Express.

[18] Gallego S., Márquez A., Marini S., Fernández E., Ortuño M., Pascual I. (2009). In dark analysis of PVA/AA materials at very low spatial frequencies: Phase modulation evolution and diffusion estimation. Opt. Express.

[19] Gallego S., Márquez A., Ortuño M., Francés J., Marini S., Beléndez A., Pascual I. (2011). Surface relief model for photopolymers without cover plating. Opt. Express.

